# Synthesis of ZIF-8/Fly Ash Composite for Adsorption of Cu^2+^, Zn^2+^ and Ni^2+^ from Aqueous Solutions

**DOI:** 10.3390/ma13010214

**Published:** 2020-01-04

**Authors:** Caili Wang, Runquan Yang, Huaifa Wang

**Affiliations:** 1 College of Mining Engineering, Taiyuan University of Technology, Taiyuan 030024, China; yangrunquan@tyut.edu.cn (R.Y.); wanghuaifa@tyut.edu.cn (H.W.); 2 State Environmental Protection Key Laboratory of Mineral Metallurgical Resources Utilization and Pollution Control, Wuhan University of Science and Technology, Wuhan 430081, China

**Keywords:** fly ash particles, ZIF-8, adsorption, heavy metal ions, nanomaterials

## Abstract

In this study, fly ash (FA) coated with ZIF-8 (ZIF-8/FA) nanocomposite was first synthesized by taking 2-methylimidazole and zinc nitrate hexahydrate as reactants and then used as an adsorbent for adsorption of copper, zinc, and nickel ions from aqueous solution. The characteristic of FA and ZIF-8/FA samples were analyzed based on the data from scanning electronic microscopy (SEM), energy dispersive spectrometer (EDS), X-ray diffraction (XRD), Fourier transform infrared (FTIR) spectroscopy, grain size analyzer and N_2_ adsorption-desorption isotherm. The results showed that ZIF-8 deposited on the FA evenly. The average crystallite size of ZIF-8 on the surface of FA is 15.85 nm. The specific surface area of FA was increased from 1.8 to 249.5 m^2^/g. The adsorption efficiency of the ZIF-8/FA nanocomposite for the removal of heavy metal ions from aqueous solution was optimized in terms of different parameters such as pH, adsorbent dosage, and contact time. It was shown that the saturated adsorption amounts of the obtained composite for adsorption of Cu^2+^, Zn^2+^, and Ni^2+^ are 335, 197, and 93 mg·g^−1^. ZIF-8/FA had better stability and more mesoporous volume than that of ZIF-8 and exhibited higher rate for adsorption of heavy metal ions from aqueous solution than FA and ZIF-8, suggesting an adsorption synergy between ZIF-8 and FA. The adsorption mechanism of heavy metal ions by ZIF-8/FA includes surface adsorption, pore adsorption, and ion exchange. The obtained ZIF-8/FA nanocomposite can solve the encountered problems of FA for low adsorption and the difficult recycling of ZIF-8 for their small size, high cost, and poor stability.

## 1. Introduction

Water is the most critical resource for human life. However, nowadays, ecological environment and water ecosystem is seriously polluted by various organic and inorganic pollutants emitted from industries. The protection and treatment of water have become the most concerned issues in modern society.

Heavy metals are of most danger among various pollutants. Removal of heavy metal ions especially copper, zinc, and nickel ions with environmentally friendly and low-cost adsorbent from wastewater has attracted considerable attention recently. The reason for this is that excessive copper, zinc and nickel ions cause serious threat to the environment, animals, and humans and they are non-biodegradable and toxic [1,2,3].

Various heavy metal wastewater treatment techniques including ion exchange [4], ultra- filtration [5], chemical precipitation [6], electro-coagulation [7], reverse osmosis [8], adsorption [9], advanced oxidation [10,11], and flotation [12] have been adopted to remove copper, zinc, and nickel ions from aqueous solution. Among which, adsorption technique is recognized as the most promising one owing to its low-cost, high efficiency, flexibility in operation, and regeneration by suitable desorption process [1].

Metal-organic frameworks (MOFs) are crystal materials constructed from metal oxygen clusters or metal ions and rigid or semi-rigid organic ligands [13]. Metals and organic ligands are usually connected by covalent bonds or ionic covalent bonds. Metal-organic frame materials generally have rigid regular channel or cage structure. Since the components contain organic parts, the directional parts of organic synthesis can be transferred to the synthesis of organic skeleton compounds. Therefore, organic materials with metal frameworks are easier to be designed, synthesized, regulated, and modified. Since MOFs has the common characteristics of both organic polymer and inorganic compounds, many researchers began to pay attention to it. MOFs has the characteristics of low density, high specific surface area, structural-functional design, adjustable channel size, etc., [14]. Therefore, it has great application potential in gas adsorption and separation [15], ion exchange [16], chemical catalytic process [17], and gas storage [18].

The American Yaghi team developed and prepared the ZIF-8 material in the early 21st century. They dissolved zinc nitrate hexahydrate (Zn(NO_3_)_2_·6H_2_O) and 2-methylimidazole (2-mlM) in n-n dimethyl formamide (DMF), respectively, and prepared zeolitic imidazolate frameworks (ZIFs) with chemical formula of Zn(mIM)_2_·(DMF)·(H_2_O)(ZIF-8) by solvent thermal method. The compounds with zeolite sodalite topology structure, namely the transition metal zinc to replace Al or Si in molecular sieve as four nodes, 2-methyl imidazole and its derivatives as ligands, to replace the molecular sieve in O to synthesize a new kind of metal organic framework compound ZIF-8. ZIF-8 has the advantages of large specific surface area, porous, adjustable properties, etc., as most MOFs materials. Zn_4_ (MeIM)_4_ ring and Zn_6_ (MeIM)_6_ ring formed ZIF-8 sodalite structure, the effective diameter is about 12.6 A, the ring cage has a relatively large specific surface area about 1630 m^2^/g, the pore volume is 0.63 m^3^/g. ZIF-8 material has a relatively typical macroporous cage small aperture structure, which effectively illustrates it with great adsorption capacity [19]. ZIF-8 is one of the most widely used MOFs materials of the current research, the research on its application involves gas adsorption, separation, hydrogen storage and catalysis, and other fields. Compared with other MOFs materials, ZIF-8 has a certain hydrothermal stability [20], which ensures that it can be used in water treatment and other fields.

Zeolite-imidazolate frameworks (ZIFs) used as adsorbents to remove copper ions from wastewaters due to large surface area, multiple active sites and high porosity compared with other conventional inorganic porous materials have become a main research focus [21], nevertheless they are relatively expensive. In addition, ZIFs have high surface energy owing to the small size, thus leading to their poor stability and difficulty in recycling and separating from aqueous solution. Depositing ZIFs onto low-cost and easily available adsorbents of large sizes to obtain composite materials is considered as an applicable method to improve their stability, make the separation easier, and reduce the cost [22].

To date, hundreds of low-cost adsorbents have been published for the heavy metal wastewater treatment [23,24,25]. Among which, FA has attracted great attention for its low cost and easy availability. However, the low adsorption capacity and efficiency of fly ash limit its application [23].

Combining metal-organic frameworks (MOFs) with a variety of functional materials have received considerable attention recently because of their enhanced performances and widespread applications. Therefore, it is assumed to solve the problems of FA and ZIFs by depositing nano ZIFs onto the surface of FA to prepare composite material.

In the present paper, ZIF-8 was coated on the surface of FA by taking zinc nitrate hexahydrate and 2-methylimidazole as reactant to solve the encountered the problems of FA for low adsorption and the difficulty for recycling of ZIF-8 for their small size, high cost, and poor stability. The characteristics of the as-synthesized product was discussed and analyzed based on the data from SEM, EDS, XRD, BET, FTIR, grain, and pore size distribution and used as a new and efficient adsorbent to remove copper, zinc, and nickel ions from aqueous solution.

## 2. Materials and Methods

### 2.1. Materials

The FA powder was provided by ShangHai GeRuiYa Nano Materials Co. Ltd. The chemical composition of FA sample is as follows: SiO_2_: 54.70 mass%, Al_2_O_3_: 29.78 mass%, Fe_2_O_3_: 4.06 mass%, TiO_2_: 1.25 mass%, CaO: 3.30 mass%, and LOI: 3.26%, in which SiO_2_ and Al_2_O_3_ accounted for more than 80%, suggesting that FA has stronger activity. Zn(NO_3_)_2_·6H_2_O (≥99.0%), 2-methylimidazole (≥98.0%), Ni(NO_3_)_2_.6H_2_O (≥99.0%), Cu(NO_3_)_2_·3H_2_O (≥99.0%), and methanol (≥99.7%) were obtained from Sigma-Aldrich. All chemicals and reagents were of analytical grade without further purification.

### 2.2. Synthesis of ZIF-8/FA

ZIF-8/FA sample was synthesized in the following way. In the whole process, the rotate speed of magnetic stirring apparatus was kept at 500 rpm, and the temperature was kept as 298 K. First, 1 g of zinc nitrate hexahydrate (Zn(NO_3_)_2_·6H_2_O) was added into 50 mL of methanol and stirred continuously for 30 min. Then, 1 g of FA was added into the above solution and stirred continuously for 60 min. Next, 3 g of 2-methylimidazole (2-mIM) was added into 50 mL methanol and stirred continuously for 30 min. Finally, the above 2-methylimidazole solution was added to the FA and zinc nitrate hexahydrate mixed solution and stirred continuously for 18 h. After that, the product was collected by centrifugation (7800 rpm, 10 min), washed three times by 180 mL of methanol and dried at 333 K for 24 h. The as-synthesized sample was marked as ZIF-8/FA.

### 2.3. Characterization of Materials

The morphologies of FA and ZIF-8/FA samples were taken with a JSM-7001F scanning electron microscope (SEM) (Japan Electron Optics Laboratory Co., LTD, Akishima, Japan). X-ray diffraction patterns (XRD) of FA and ZIF-8/FA samples were performed on a MiniFlex600 diffractomer (Japan Rigaku Co., Ltd., Akishima, Japan) with Cu Kα radiation (50 KV, 200 mA). The specific surface areas of FA, ZIF-8/FA, and pure ZIF-8 were calculated with the Brunauer-Emmet-Teller (BET) method by ST-2000 nitrogen sorption isotherm (Beijing Beifen Instrument Technology Co., Ltd., Beijing, China) measurement. The samples were degassed at 120 °C overnight before measurement with N_2_ adsorption-desorption isotherms. Total pore volume (V_t_) was determined by the volume of liquid nitrogen adsorbed at 0.99 (P/P_0_). The micropore volume (V_micro_) was calculated using t-plot method and mesopore volume (V_meso_) was obtained by subtracting V_micro_ from V_t_. Fourier transform infrared spectroscopy (FTIR) spectra of FA and ZIF-8/FA samples were undertaken on a TENSOR27 spectrometer (German Bruker Co., Ltd., Karlsruhe, German) in the range of 4000–500 cm^−1^. The particle size distributions of FA and ZIF-8/FA samples were examined with a BT-1500 decanter grain-size distribution meter (Dandong, Baite Instrument Co., Ltd., Dandong, China).

### 2.4. Adsorption Experiments

The adsorption studies were carried out by mixing a certain amount of adsorbent and a certain volume of copper, zinc, and nickel ions solutions with the magnetic stirrer at room temperature (298 K), respectively. The initial concentrations for copper, zinc, and nickel ions solutions were around 100 mg/L and the amount of adsorbent was in the range of 0.1–1.5g/L. The magnetic stirrer was operated at 400 rpm to make adsorbent dissolved adequately. 240 min were used as the equilibrium time. All the solution was filtered by centrifugation (7800 rpm, 10 min) to remove the adsorbent at the given time and 7 mL filtered solution of each sample was taken out from the bottle for finally analyzing the concentration of copper, zinc, and nickel ions by ICP-OES. The amount of copper, zinc, and nickel ions adsorbed (*Q*e) and the removal percentage (%Removal) of copper, zinc, and nickel ions could be calculated with the following equations:

(1) %Removal=(Co−CtCo)×100

(2) $$Qe=\frac{(Co-Ce)V}{m}$$

where *C_O_* is the initial concentration of copper, zinc, and nickel ions, *C_t_* (mg/L) is the concentration of copper, zinc, and nickel ions at time *t*. *Ce* (mg/L) is the concentration of copper, zinc and nickel ions at equilibrium, *V* (L) is the volume of solution, and *m* (g) is the weight of the adsorbent.

## 3. Results and Discussion

### 3.1. Morphology

**Figure 1:**
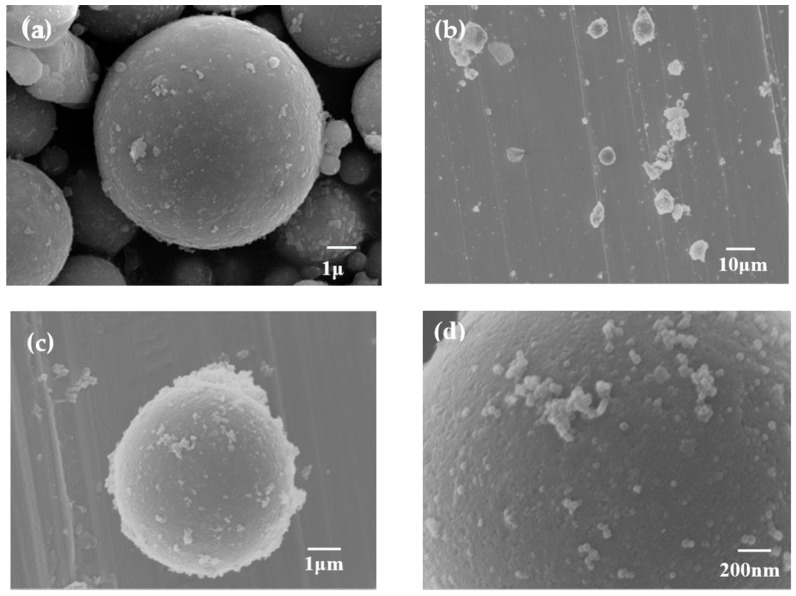

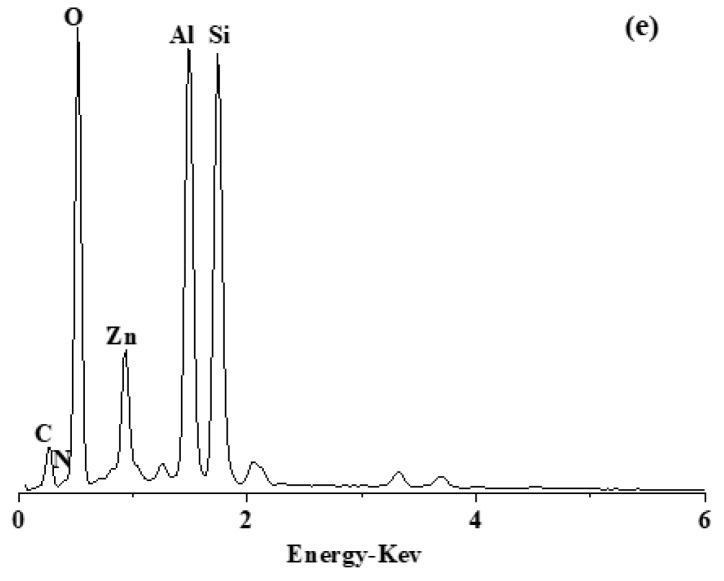
Scanning electron microscopy (SEM) images of (**a**) fly ash (FA), (**b**–**d**) ZIF-8/FA composite and (**e**) energy dispersive spectrometer (EDS) of ZIF-8/FA composite.

Figure 1 shows the SEM micrographs of FA and ZIF-8/FA samples and EDS spectra of ZIF-8/FA composite. It can be seen that the morphology of FA shows spherical shape (Figure 1a). A rough surface with nanosize particles could be clearly observed for ZIF-8/FA (Figure 1d), suggesting the formation of ZIF-8 onto the surface of FA. Moreover, as shown in Figure 1b,c, the surface of FA was fully covered by ZIF-8 particles, and no bare surface could be seen, revealing that the composite with well distributed ZIF-8 nanoparticles on the FA surface. This conclusion could also be confirmed by EDS spectra of ZIF-8/FA composite (Figure 1e).

### 3.2. Grain Size Distribution

It is necessary to analyze the grain size distribution to determine the deposition of ZIF-8 on the FA. The grain size distributions of FA and ZIF-8/FA samples are as follows. For FA, D_3_ = 8.20 µm, D_10_ = 11.66 µm, D_25_ = 16.76 µm, D_50_ = 24.32 µm, D_75_ = 37.17 µm, D_97_ = 60.53 µm, while for ZIF-8/FA, D_3_ = 8.61 µm, D_10_ = 11.94 µm, D_25_ = 17.13 µm, D_50_ = 25.12 µm, D_75_ = 39.95 µm, D_97_ = 63.11µm. The grain size of ZIF-8/FA is bigger than that of FA, indicating that all the nano ZIF-8 particles deposited on the surface of FA, confirming the SEM results.

### 3.3. XRD Analysis

**Figure 2:**
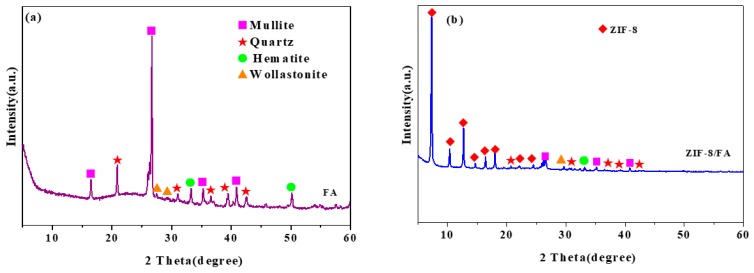

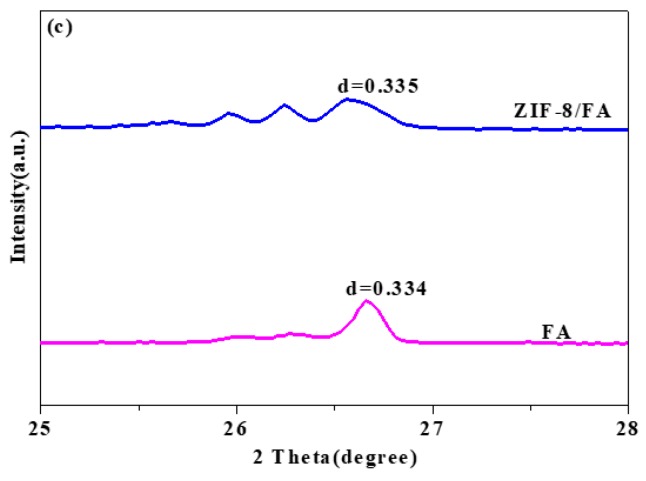
X-ray diffraction (XRD) spectrum of (**a**) FA and (**b**), (**c**) ZIF-8/FA composite.

XRD patterns of FA and ZIF-8/FA samples are shown in Figure 2. It can be seen from Figure 2a that the FA raw material contains a large number of amorphous and crystalline substances, and the main crystal substances are mullite Al_6_Si_2_O_13_ (No.150776) and quartz SiO_2_ (No.461045). The characteristic peaks of the crystal phases of quartz and mullite are very significant, indicating that the crystallinity of the two is very good, which is consistent with the characteristic diffraction peaks reported in the literature [26]. The crystallite reflexes of hematite (No.521449) and wollastonite (No.420550) are also observed in Figure 2a. As shown in Figure 2b, after coating with ZIF-8, both peaks representing the FA phase and ZIF-8 phase could be found in the composite, indicating successful synthesis of the ZIF-8/FA composite. The (001), (002), (112), (022), (013), (222), (114), (233) peaks of ZIF-8 samples are obviously observed at 2θ = 7.33°, 10.41°, 12.75°, 14.73°, 16.43°, 18.07°, 22.17°, and 24.54°, respectively [21]. Figure 2c shows that the change in the *d* spacing of FA after coating with ZIF-8 is negligible and these signify the ZIF-8 nanoparticles are on the surface of FA. The average crystallite size of ZIF-8 on the surface of FA calculated by Scherrer equation (D = Kλ/(βcosθ) is 15.85 nm, where β = 0.00916 is the full width half maximum(FWHM) of the sample and *θ* is the diffracting angle, K = 0.94 is a coefficient, λ = 0.15418 nm is the X-ray wavelength [27].

### 3.4. FTIR Analysis

**Figure 3:**
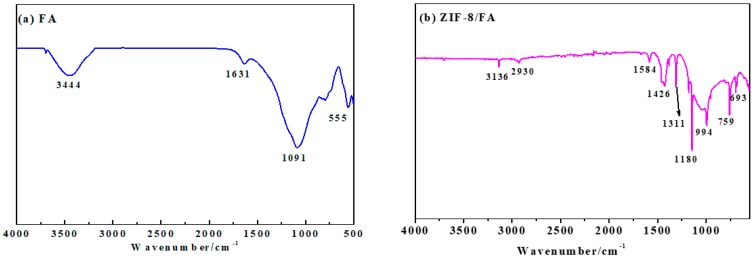
Fourier transform infrared (FTIR) spectra of (**a**) FA and (**b**) prepared ZIF-8/FA composite powders.

Figure 3 is the FTIR of FA and ZIF-8/FA powders. The bands at 500–555cm^−1^ were ascribed to Al–O stretching vibration and Si–O bending vibration of FA [27]. The bands at 1091 cm^−1^ corresponded with the Si–O–Si asymmetric stretching vibration adsorption peak [27]. The bands at 3444 cm^−1^ and 1631 cm^−1^ were attributed to the O–H stretching and bending vibration adsorption peaks of FA, respectively [27] (Figure 3a).

**Figure 4:**
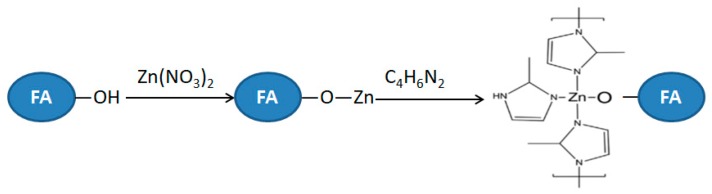
Preparation mechanism of ZIF-8/FA composites.

After coated with ZIF-8 (Figure 3b), both peaks representing the FA and ZIF-8 could be found in the composite. New infrared diffraction peaks at 3136, 2930, 1584, 1426 cm^−1^ are feature peaks of imidazole, and the infrared diffraction peak at 500 cm^−1^ is the peak of Zn-O telescopic vibration, which is consistent with the earlier published papers [28,29]. Moreover, the band at 3444 cm^−1^ disappeared and C-H stretching vibration adsorption peak of methyl and imidazole ring appeared in 3136 and 2930 cm^−1^ [28], respectively. The O–H bending vibration adsorption peak at 1631 cm^−1^ disappeared and C = N stretching vibration adsorption peaks appeared at 1584 cm^−1^. The bands at 1180 and 994 cm^−1^ show the C-N stretching vibration adsorption peaks [28]. In addition, the peaks at 1350–1500 cm^−1^ belong to the entire ring stretching. The in-plane bending of the ring are found at 900–1350 cm^−1^ while those below 800 cm^−1^ are associated with the out-of-plane bending of the ring [29]. Considering the above analysis, the preparation mechanism of ZIF-8/FA composites can be explained by Figure 4 [30].

### 3.5. Specific Surface Area and Pore Size

**Figure 5:**
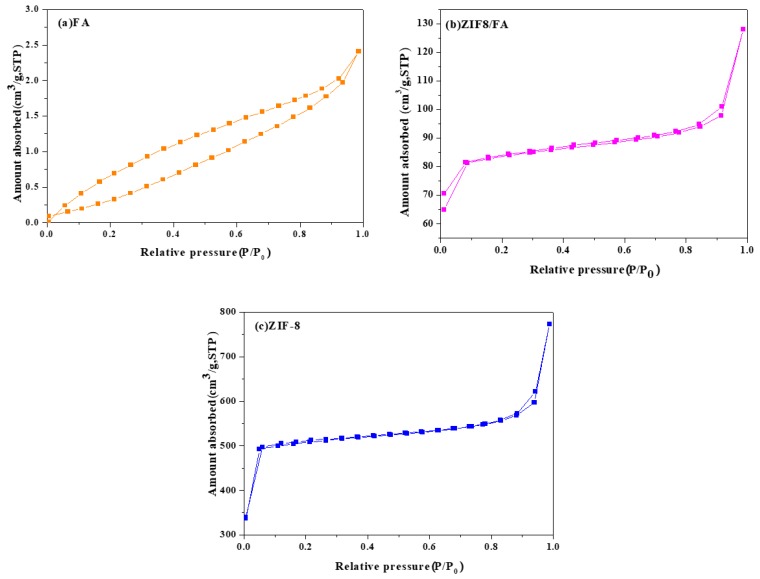
Nitrogen adsorption–desorption isotherms of (**a**) FA, (**b**) ZIF-8/FA, and (**c**) ZIF-8.

**Figure 6:**
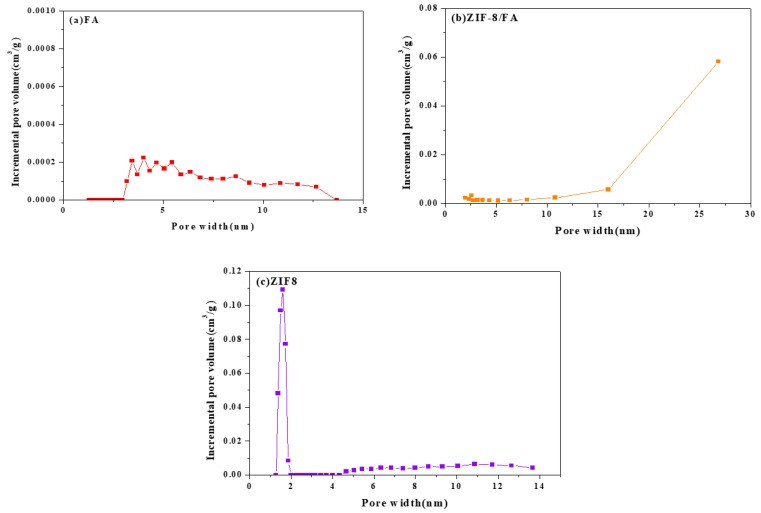
Pore size distribution of (**a**) FA, (**b**) ZIF-8/FA, and (**c**) ZIF-8.

Figure 5 and Figure 6 and Table 1 are the nitrogen adsorption–desorption isotherms, pore size distribution, BET specific surface area, average grain size of (a) FA, (b) ZIF-8/FA, and (c) ZIF-8 samples, respectively. It can be seen that FA sample shows a typical IV adsorption isotherm with a H3-type hysteresis loop, which may be attributed to the relatively large mesopores, confirming from Figure 6a. Table 1 shows that FA has no micropores (0 cm^3^/g) and low volume of mesopores (0.0025 cm^3^/g). The specific surface area of FA is only 1.8 m^2^/g, while those of ZIF-8 reaches 1594.8 m^2^/g. Pure ZIF-8 shows a typical type I isotherm (Figure 5c) and characteristic of microporous materials with high volume of micropores (0.67 cm^3^/g) and low volume of mesopores (0.06 cm^3^/g),confirming from Table 1 and Figure 6c. Unlike FA and ZIF-8, ZIF-8/FA composite displays a combination of type I and type IV isotherm with a significant hysteresis, suggesting coexistence of microporous and mesoporous structure in the composite, which is confirmed from Figure 6b. The micropore is convinced from the inheritance of porous ZIF-8. ZIF-8/FA composite is much superior in surface area compared with FA, with a value of 249.5 m^2^/g, and it not only contains micropores less than 2 nm, but also includes more volume (0.078 cm^3^/g) of larger mesopores about 2–27 nm, which is higher than that of FA and ZIF-8 (Figure 6b).

### 3.6. Adsorption Ability

#### 3.6.1. Comparison of FA, ZIF-8/FA, and ZIF-8

**Figure 7:**
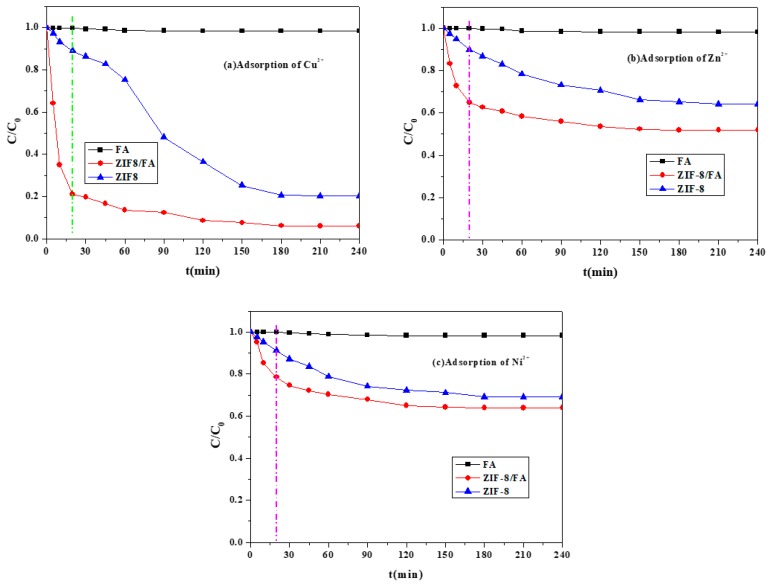
Adsorption of Cu^2+^, Zn^2+^, and Ni^2+^ using different samples (Adsorbent = 50 mg, (**a**) Cu^2+^ = 50 mL of 100 mg/L, (**b**) Zn^2+^ = 50 mL of 100 mg/L, (**c**) Ni^2+^ = 50 mL of 100 mg/L).

The adsorption results of FA, ZIF-8/FA, and pure ZIF-8 for removal of Cu^2+^, Zn^2+^, and Ni^2+^ are shown in Figure 7. It can be seen that after 4 h adsorption (the adsorption reached equilibrium), only 1.7 ± 0.2% (1.8 ± 0.3%, 1.6 ± 0.2%) and 79.8 ± 0.6% (35.9 ± 0.4%, 30.9 ± 0.3%) of copper (zinc, nickel) ions were adsorbed by FA and ZIF-8, respectively. Remarkably, more than 94.0 ± 0.5% (48.2 ± 0.4%, 36.1 ± 0.3%) of that could be adsorbed by ZIF-8/FA, revealing an advantageous synergy of adsorption between ZIF-8 and FA. Experiments also indicate that in the initial 20 min, ZIF-8/FA had faster adsorption rate of Cu^2+^, Zn^2+^, and Ni^2+^ than FA and pure ZIF-8.

Based on the above results, it could be deduced that the adsorption of copper, zinc, and nickel ions onto ZIF-8/FA was enhanced because of its high specific surface area, which provided more active sites for the interaction of copper, zinc, and nickel ions, resulting in a high adsorption activity. Besides, more mesopores and active sites became available in the highly dispersed ZIF-8 on FA, which facilitates the adsorption, thus improving the adsorption rate toward copper, zinc, and nickel ions removal. This could also be explained by the research works from Sayari [24] and Bibby [25]. Their studies showed that, with the increase of mesopore size, the material had faster adsorption for Cu^2+^, Zn^2+^, and Ni^2+^, while for materials with smaller mesopore size, the adsorption abilities could not be fully developed due to steric hindrance.

The concentration of Zn^2+^ in the filtered solution discharged by ZIF-8 or ZIF-8/FA was analyzed to evaluate its stability. The concentration of Zn^2+^ discharged by pure ZIF-8 for adsorption of Cu^2+^, Zn^2+^, and Ni^2+^ is 77, 30, and 29mg/L, respectively, while that discharged by ZIF-8/FA is 3.6, 2.2, and 1.4 mg/L, respectively. The reason for this is that Si-O-Zn bonds formed between FA and ZIF-8, the Cu^2+^, Zn^2+^, and Ni^2+^ would be adsorbed by the active site in the organic groups covered on the surface of the composite, preventing the ion exchange reaction between Zn^2+^ and Cu^2+^ or Ni^2+^, while for pure ZIF-8, the Zn bared in the solution, the ion exchange reaction between Zn^2+^ and Cu^2+^ or Ni^2+^ occurred, leading to the higher concentration of Zn^2+^ in the filtered solution. All the data confirmed that ZIF-8/FA has better stability than pure ZIF-8. Therefore, in the following experiment, only the adsorption ability of ZIF-8/FA on heavy metal ions was conducted.

The adsorption tests containing mixtures of Cu^2+^, Zn^2+^, and Ni^2+^ by ZIF-8/FA were also investigated. The initial concentration of Cu^2+^, Zn^2+^ and Ni^2+^ is 33.33, 33.33, 33.33 mg/L, respectively. The volume is 50 mL, the adsorbent dosage of adsorbent is 50 mg. The adsorption time is 4 h. The equilibrium concentration of Cu^2+^, Zn^2+^, and Ni^2+^ after adsorbed by ZIF-8/FA was tested to be 0.04, 34.18, 32.43 mg/L. It can be seen that copper ions were completely adsorbed on ZIF-8/FA, while zinc and nickel ions could not be adsorbed for the interference of competing ions.

#### 3.6.2. pH Value

**Figure 8:**
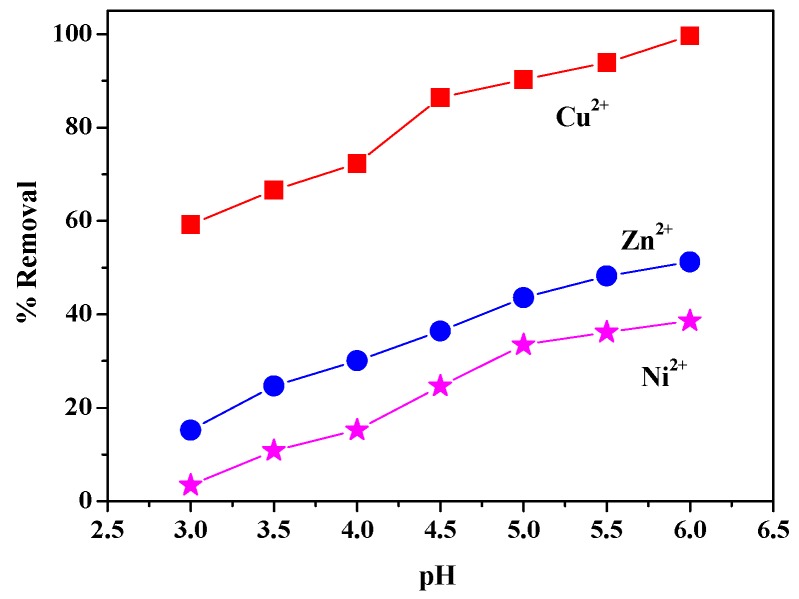
Effect of pH of heavy metal ions solution on removal rate (adsorbent:1 g/L, heavy metal ions concentration:100 mg/L, 298 K, 4 h).

The relationship between removal rate of the ZIF-8/FA and the pH value is presented in Figure 8. In the preliminary test, we found that when pH value of the aqueous mixture is less than 3, some of the N-Zn bonds of ZIF-8/FA will break, while pH > 6, the heavy metal ions will precipitate with the free anion OH^−^ in the aqueous solution. Therefore, these two ranges are not suitable for the study of ZIF-8/FA on heavy metal ions adsorption process. So the pH of the aqueous solution in the range of 3–6 was investigated. It can be seen from Figure 8 that the removal rate of heavy metal ions increases with the increase of pH value of the aqueous solution. With the increase of pH value of aqueous solution, the electrostatic revulsion among ZIF-8/FA surface and heavy metal ions decreased. When pH is low, the free H^+^ occupies the active site on the outer surface of ZIF-8/FA. By the increase of pH value, the H^+^ concentration decreased, the competitive relationship weakened, and the adsorption of heavy metal ions on the active site becomes larger, leading to an increased removal rate of heavy metal ions. Considering the above analysis, the pH value was selected as 5.5 in the next parallel experiment.

#### 3.6.3. ZIF-8/FA Concentration

**Figure 9:**
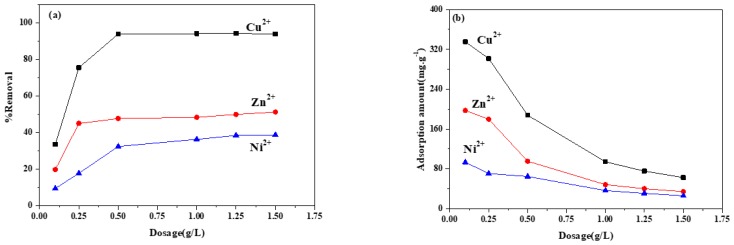
Effect of ZIF-8/FA dosage on (**a**) removal rate and (**b**) adsorption amount of heavy metal ions (heavy metal ions concentration:100 mg/L, 298K, 4 h, pH:5.5).

Figure 9 shows the effect of ZIF-8/FA dosage on the removal rate and adsorption amount of heavy metal ions. It can be seen that the removal rate increases with the increase of the ZIF-8/FA dosage, while the adsorption amount decreases with the rise of adsorbent concentration. The reason is that the adsorption active site increases with the increase of adsorbent concentration while the heavy metal ions is limited, thus leading to the decrease of adsorption amount. When the adsorbent concentration is very low (0.1 g/L), the adsorption amount of ZIF-8/FA is close to the saturated adsorption capacity. Therefore, the saturated adsorption capacities of the ZIF-8/FA on Cu^2+^, Zn^2+^, and Ni^2+^ were 335, 197, and 93 mg/g, respectively.

The comparison of the saturated adsorption capacities of copper, zinc and nickel ions with different adsorbents is listed in Table 2. It can be seen that ZIF-8/FA has a relatively large adsorption capacity of 335, 197, and 93 mg.g^−1^ for Cu^2+^, Zn^2+^, and Ni^2+^, respectively, indicating that ZIF-8/FA is a good candidate for the removal of Cu^2+^, Zn^2+^, and Ni^2+^ from aqueous solutions.

### 3.7. Recycling Study of ZIF-8/FA Composites

**Figure 10:**
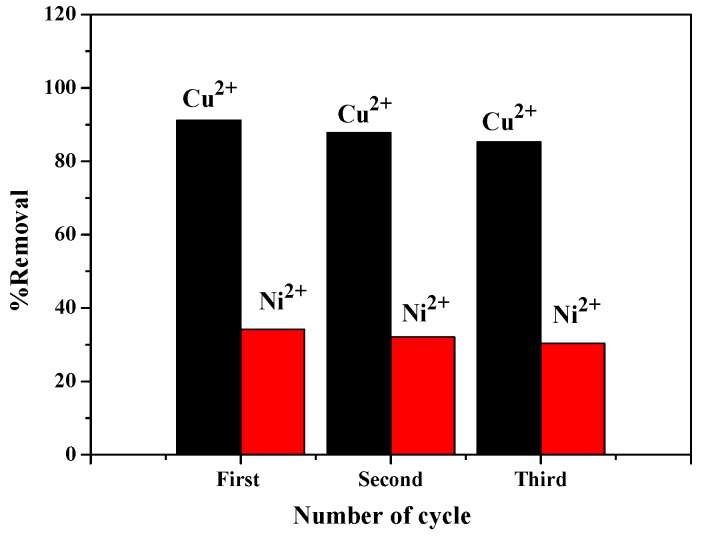
Recyclability of ZIF-8/FA composite for adsorption of heavy metal ions.

ZIF-8/FA composites were studied for the recycling experiments as reusability is an important factor for an adsorbent in practical application. Cu^2+^ and Ni^2+^ were used as representatives for recycling study (Figure 10). For cyclic runs, ZIF-8/FA composites were separated by centrifugation after the adsorption process and washed with 0.1 mol/L HCl and deionized water and dried for regeneration. Through these simple processes, the adsorbed Cu^2+^ and Ni^2+^ were washed out and the ZIF-8/FA composites were regenerated. Figure 10 is the recyclability of ZIF-8/FA composite for adsorption of heavy metal ions. It can be seen that the adsorption percentage of Cu^2+^ with ZIF-8/FA composites remained above 85% after three times of recycling test (91.2% for n = 1, 87.8% for n = 2, and 85.3% for n = 3) while that of Ni^2+^ with ZIF-8/FA composites remained above 30% after three times of recycling test (34.2% for n = 1, 32.1% for n = 2, and 30.4% for n = 3). Results of the recovery tests indicated that ZIF-8/FA composites can be employed as a potential adsorbent for the removal of heavy metal ions.

### 3.8. Adsorption Mechanism

Considering the above analysis and some research work reported [37], it is speculated that the adsorption mechanism of Cu^2+^, Zn^2+^, and Ni^2+^ by ZIF-8/FA includes: surface adsorption, pore adsorption, and ion exchange.

## 4. Conclusions

In this work, ZIF-8 with average crystallite size 15.85 nm was successfully deposited as a coating shell on the surface of FA. The synthesized composite exhibited higher adsorption capacity and faster rate for copper, zinc, and nickel ions compared with FA and the pure ZIF-8, which is attributed to the high volume of mesopores, more active sites, and large specific surface area. Moreover, ZIF-8/FA had better stability than pure ZIF-8. The composites could not only solve the encountered problems of FA for low adsorption but also the difficulty for recycling of ZIF-8 for their small size, high cost, and poor stability. Therefore, the ZIF-8/FA composite is an excellent candidate for adsorption of copper, zinc, and nickel ions from wastewater.

## Figures and Tables

**Table 1:** Specific surface area and porosity of FA, ZIF-8 and ZIF-8/FA composites.

Sample	S_BET_ (m^2^g^−1^)	V_t_ (cm^3^g^−1^)	V_micro_ (cm^3^g^−1^)	V_meso_ (cm^3^g^−1^)	Average Pore Diameter (nm)
FA	1.8	0.0025	0	0.0025	4.21
ZIF-8/FA	249.5	0.08	0.002	0.078	11.29
ZIF-8	1594.8	0.73	0.67	0.06	4.22

**Table 2:** Comparison of adsorption capacities (mg·g−1) of heavy metal ions using different adsorbents.

Adsorbent Type	Saturated Adsorption Capacities (mg.g^−1^)			References
	Cu^2+^	Ni^2+^	Zn^2+^	
G-ZnO composite	37.54			[31]
Modified Carbons		9.81	10.7	[32]
ZnO/activated carbon cloth	1300			[33]
Nanometer-size TiO_2_	26.5			[34]
Tourmaline		30.67	37.88	[35]
Indonesian peat			32.07	[36]
Fly ash derived geopolymer	90			[3]
ZnO-Gr		66.7		[2]
ZIF-8/FA composite	335	93	197	This work
